# The normal and fibrotic mouse lung classified by spatial proteomic analysis

**DOI:** 10.1038/s41598-022-12738-9

**Published:** 2022-05-24

**Authors:** Roberta Ciccimarra, Maddalena M. Bolognesi, Matteo Zoboli, Giorgio Cattoretti, Franco F. Stellari, Francesca Ravanetti

**Affiliations:** 1 Department of Veterinary Science, Università di Parma, Parma, Italy; 2 Department of Medicine and Surgery, Università di Milano-Bicocca, Monza, Italy; 3 Corporate Preclinical R&D, Chiesi Farmaceutici S.P.A., Largo Belloli 11/A, 43122 Parma, Italy

**Keywords:** Experimental models of disease, Imaging techniques, Bioinformatics, Proteomics, Mouse, Immunohistochemistry, Anatomy, Respiratory tract diseases, Chronic obstructive pulmonary disease, Chronic inflammation

## Abstract

Single cell classification is elucidating homeostasis and pathology in tissues and whole organs. We applied in situ spatial proteomics by multiplex antibody staining to routinely processed mouse lung, healthy and during a fibrosis model. With a limited validated antibody panel (24) we classify the normal constituents (alveolar type I and II, bronchial epithelia, endothelial, muscular, stromal and hematopoietic cells) and by quantitative measurements, we show the progress of lung fibrosis over a 4 weeks course, the changing landscape and the cell-specific quantitative variation of a multidrug transporter. An early decline in AT2 alveolar cells and a progressive increase in stromal cells seems at the core of the fibrotic process.

## Introduction

Single cell biology, brought to fruition by advances in gene sequencing and computational progress, has revolutionized how we understand biological processes in health and in pathology^1^.

Applying these techniques to the analysis of individual cells in-situ, i.e. within the tissue microenvironment, has added the information of the tissue sociology of the specimen, answering to the growing need to investigate it, due to various cellular functions, the spatial organization of molecular targets, the relationship among multiple cell types and morphology.

While in situ single cell sequencing can provide information on the RNA species in the thousands, in situ multiplex staining has a limit of around 100 antibodies, in general 30–60 for the time being^2^. To distinguish the techniques which can add antibodies in the dozens from the other which provide a much limited number (5–6), we will refer to the former as “high-plex” multiplexing techniques.

Single cell biology via high-plex in situ staining can be accomplished both in fresh frozen tissue sections and in sections from routinely processed tissue (formalin fixed, paraffin embedded; FFPE), depending on the technology used. FFPE material has several advantages, including the capability to create high density arrays containing several different samples, the tissue microarray technology (TMA)^3^. Being the high-plex technology complex and time-consuming by itself, the combination with the TMA technology allows an enormous, detailed analytical power in the basic and applied science field.

Murine models are an extremely helpful, standard companion to the research on human subjects in all fields of medicine. All kinds of diagnostic and therapeutic approaches used on humans have been miniaturized and applied to mice^4^, including histopathologic and phenotypic examination. The problem of using antibody-based high-plex methods on mouse tissue when using primary antibodies raised in mice is the cross reactivity with endogenous immunoglobulins: we have overcome this problem by using anti-isotype secondary Abs^5^ on FFPE mouse tissue, therefore we have broadened the antibody portfolio which can be used on mouse histopathology.

Mouse specimens have been extensively used for single cell biology studies in all fields, largely via single cell RNA sequencing, rarely for in situ spatial proteomics and high-plex staining.

We have applied the MILAN high-plex technique^6^ to a murine model of Bleomycin-induced (BLM) lung fibrosis. Although, this animal model is the most used in preclinical study and the best characterized either to investigate lung fibrotic mechanisms or to screen drugs, and the American Thoracic Society (ATS) has suggested recommendations for preclinical assessment of antifibrotic compounds, a deep molecular profiling characterization is still lacking^7–9^.

Bleomycin elicits a time-dependent lung fibrotic process^10^, which has several similarities with the human counterpart^11^. The BLM delivered through the airways determines bronchiolocentric accentuated fibrotic changes through a multistep injury starting with epithelial cell damage (in mice, days 1–3) followed by acute interstitial and intra-alveolar inflammation (days 3–10) and ultimately to fibroblasts activation and remodeling of extracellular matrix leading to fibrosis, extracellular matrix deposition (days 10–21 with a peak around day 14)^12^. In mice, the alterations induced by Bleomycin are heterogeneous, time-limited and self-resolving, with the drawback of a narrow time window for preclinical testing. In fact, histological analysis revealed fibrosis pattern started from day 7, mainly as single fibrotic masses, and evolved at days 14 as confluent conglomerates of substitutive collagen, which last till day 21 with a tendency to resolve already at 28 day^13^.

To better understand lung fibrosis development, highplex technology has been used to detect target of interest, applying it to routinely processed tissue samples.

In this study we consider the recent observation that BLM administration induces an ABC (ATP Binding Cassette) carrier upregulation in lung, with an increasing expression of P-glycoprotein (P-gp) and transporters in C57BL/6 male mice (Park et al. 2020)^14^. The ABC carriers actively transport multiple xenobiotics across the membrane reducing the intracellular concentration of drugs and leading to a potential decrease in anti-fibrotic activity.

We present here the validation of the method and results of the single cell composition of the healthy and pathologic mouse lung, including the changes in expression of a P-gP multidrug transporter in each of the cell types identified by high-dimensional analysis.

## Results

### TMA validation

The use of tissue cores, instead of whole sections, entails higher throughput at the expense of representativeness. To address this latter aspect we used single cell analysis to validate the use of the TMA. The image segmentation strategy, centered on the identification of nuclear DAPI + containing regions of interest (ROI), approximate single-cell identification in tissue, with some limits (see supplementary methods and discussions). 23 markers + DAPI (Table 1) were used to classify all the ROI (see M&M and supplementary material).

**Table 1:** Design and antibodies specifications.

Staining round	Antigen name	Short name	Cell type	scRNAseq ref	Catalog N	Company	Species	Diluition or final concentration (µg/ml)
**Primary antibodies**								
1	Collagen1	COLL1	Stromal Cells	24 (Col1a1)	ab88147	Abcam	mo IgG3	2.5
1	Alpha smooth muscle actin	aSMA	Vasculature		1A4—M0851	Dako	mo IgG2a	0.7
2	Transgelin	SM22	Vasculature		ab10135	Abcam	goat	4
2	Desmin	DESM	Vasculature		D33—M076	Dako	mo IgG1	1.3
3	Von Willebrand Factor	vWF	Vasculature		AP22470PU-N	Origene	goat	2.5
3	Multiple Cytokeratins	PanCK	Epithelial Cells		MA1-82041	Thermo Fisher	mo IgG1	1:40
4	Mucin	MUC5b	Goblet Cells		ab77995	Abcam	mo IgG1	5
4	Macrophages Mannose Receptor	CD206	Macrophages	23 (Mrc1)	AF2535	R&D	goat	1
5	Surfactant D	SPD	AT2, alveolar progenitors	24 (Sftpd)	Sc-25324	Santa Cruz B	mo IgG1	1
5	Paired box protein 5	PAX5	B Lymphocytes		ab109443	Abcam	rb Mab	1
6	Aquaporin 5	AQP5	AT1	24	Sc-514022	Santa Cruz B	mo IgG1	1
6	Osteopontin	OPN	Macrophages, neutrophils	24	AF808	R&D	goat	1
7	Podoplanin	PDPN	AT1	24	ab11936	Abcam	s.hamster	5
7	Matrix Metalloprotease 7	MMP7	–		D4H5—3801	Cell Signaling	rb Mab	5
8	Thyroid Transcription Factor1	NKX2-1/TTF1	AT2	24 (Nkx2-1)	8G7G3—M3575	Dako	mo IgG1	1
8	T cells	CD3	T Lymphocytes		ab5690	Abcam	rb poly	0.3
9	Advanced Glycosylation End-Product Specific Receptor	RAGE	AT1	23 (Ager)	Sc-80653	Santa Cruz B	mo IgG1	1
9	Myeloperoxidase	MPO	Monocyte progenitors	24	ab9535	Abcam	rb poly	1:200
10	Homeobox only protein	HOP	AT1	24	Sc-398703	Santa Cruz B	mo IgG1	1
10	ATP-binding cassette sub-family A member 3	ABCA3	AT2	24	ab24751	Abcam	mo IgG2a	1
10	Fibroblast growth factor 10	FGF10	Stromal Cells	23	GTX30007	GeneTex	goat	1
11	Vimentin	VIM	Stromal Cells		Sc-373717	Santa Cruz B	mo IgG1	1
11	Multidrug resistance 1	P-gP	–		orb11267	Biorbyt	rb poly	1
11	Breast cancer resistance protein	BCRP	–		ab24115	Abcam	rat IgG2a	1

Name	Dilution	Catalog N						
**Secondary antibodies**								
Alexa Fluor® 488 AffiniPure Donkey Anti Goat IgG (H + L)	1:500	705-545-147						
Alexa Fluor® 488 AffiniPure Goat Anti Rabbit IgG (H + L)	1:500	111-545-144						
Alexa Fluor® 488 AffiniPure Goat Anti-Mouse IgG, Fcγ subclass 1 specific	1:200	115-545-205						
Alexa Fluor® 647 AffiniPure Donkey Anti-Goat igG (H + L)	1:150	705-005-003						
Alexa Fluor® 647 AffiniPure Goat Anti Mouse IgG, Fc subclass 1 specific	1:500	115-605-205						
Alexa Fluor® 647 AffiniPure Goat Anti Mouse IgG, Fc subclass 3 specific	1:500	115-605-209						
Alexa Fluor® 647 AffiniPure Donkey Anti Rat IgG (H + L)	1:500	712-605-153						
Rhodamine (TRITC) AffiniPure Goat Anti Mouse IgG, Fc subclass 2a specific	1:200	115-295-206						
Rhodamine (TRITC) AffiniPure Donkey Anti-Rabbit IgG (H + L)	1:200	711-295-152						

**Figure 1:**
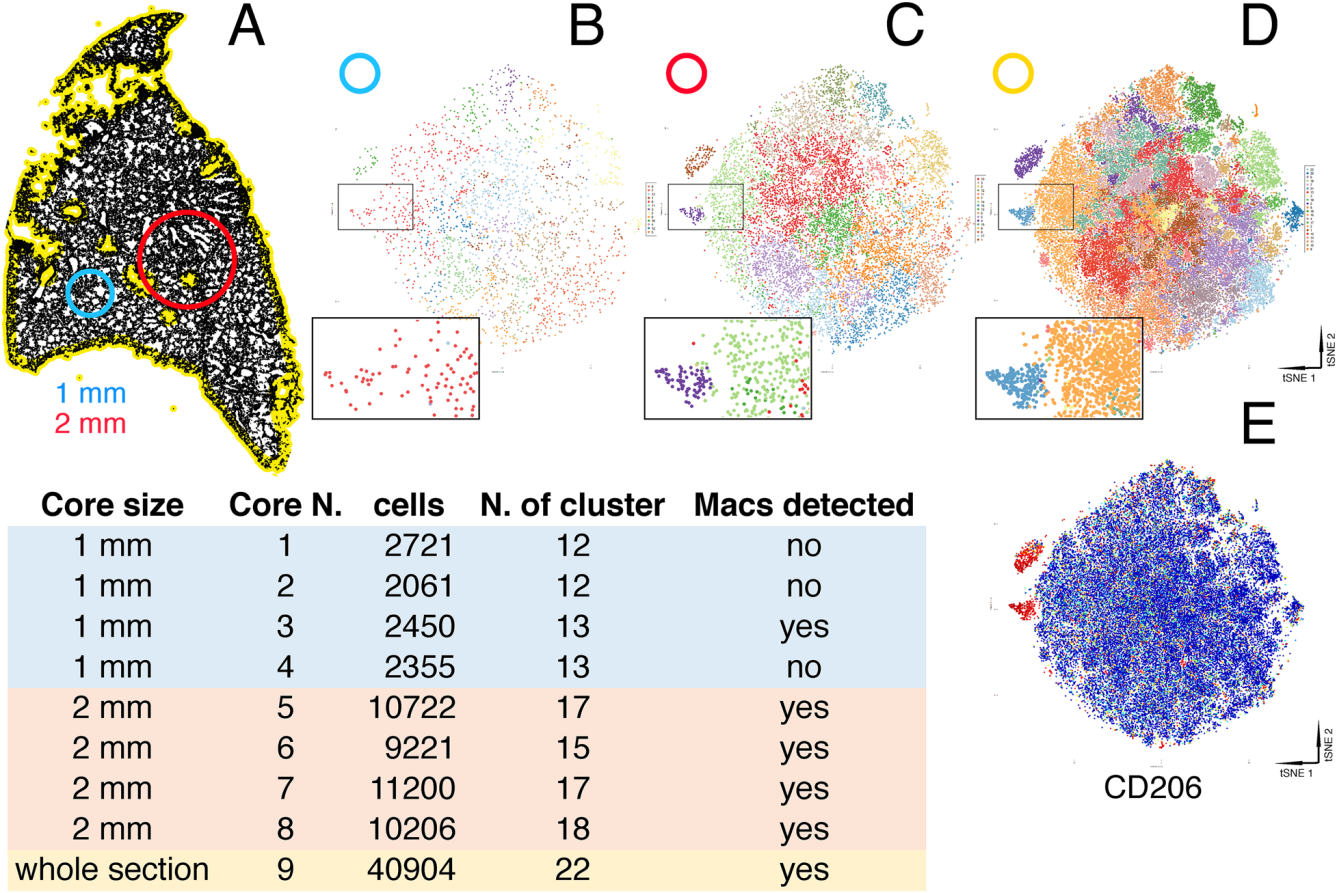
Core dimension validation for TMA construction. Representative DAPI-stained normal lung whole slide image (**A**) in which 1 (blue) and 2 (red) mm diameter representative cores are drawn. (**B**–**D**) tSNE plots derived from the 1 mm (**B**), 2 mm (**C**) cores and the whole section (**D**), from which the phenogroups have been extracted and plotted onto the tSNE plot. A detail of the plot is magnified at the bottom left of each. Note the lack of discrimination of the macrophage population from the adjacent phenogroups in the 1 mm core example, highlighted by a single color rendering. The distribution of CD206, a macrophage marker, is shown in red on the tSNE plot of the whole section (**E**). The table compares core size, cells contained, number of clusters and whether a macrophage cluster was identifiable or not.

Single cell analysis included an average of 2396 cells for 1 mm core, 10,337 for 2 mm core and 40,904 for the whole section (Fig. 1). Clusters comprising sparse cells, such as Macrophages (Fig. 1) and T cells (not shown) were equally represented in the 2 mm cores and in the whole section (Fig. 1), but ill-identified on 1 mm cores.

The 2 mm TMA cores were used for the study.

## Single cell analysis of the normal and fibrotic mouse lung

**Figure 2:**
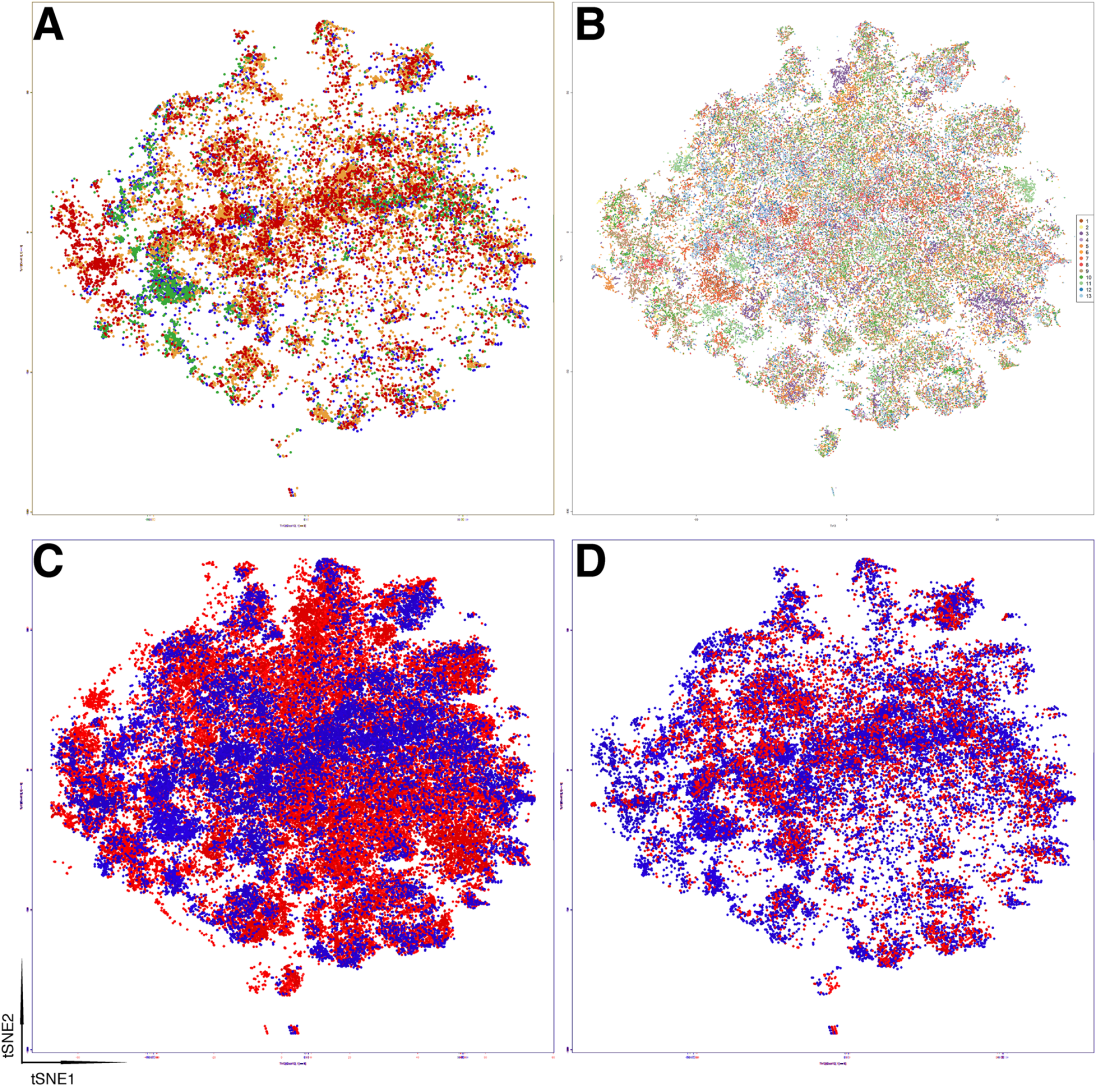
Distribution of control and treated lung cells in dimensionality-reduced 2-dimensional space (tSNE). (**A**) Four saline cases from different time points (7, 14, 21 and 28 days) are plotted according to the tSNE bidimensional coordinates, each in an unique color. Each dot represents a cell. (**B**) tSNE plot of all cases, each with an unique color. (**C**) tSNE plot of all saline samples (blue) and Bleomycin-treated samples at days 7, 14 and 21 (red). (**D**) tSNE plot of all saline samples (blue) and Bleomycin-treated samples at day 28 (red).

tSNE plots from four control animals showed a superimposition of all cells from all animals as expected (Fig. 2A). Cells from Bleomycin-treated mice were allocated in the same phenoclusters (Fig. 2C) of the controls, but with a slightly different spatial distribution for mice 7, 14 and 21 post BLM treatment (Fig. 2C). Mice at day 28th were very similar to controls (Fig. 2D). The phenoclusters contained cells from every case (Fig. 2B) demonstrating the absence of batch effect in the data.

**Figure 3:**
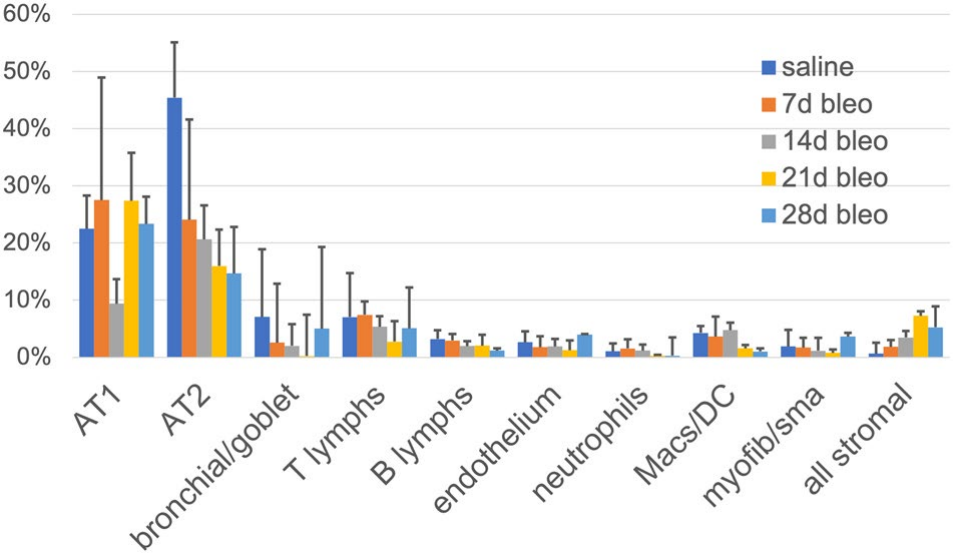
Classification and frequency of cell types in controls and treated mice. The cell types identified in the lungs are shown as mean ± SD for individual mice for each treatment. AT2 represents AT2 and transitional AT1/AT2 alveolar cells (see supplementary data); bronchial/goblet = bronchial cells and MUC5B cells merged; all stromal = phenoclusters with mesenchymal markers merged. For details see supplementary Table 1.

Phenograph clusters containing all mice from all experimental points were plotted on tSNE and a provisional cell classification was assigned to each phenogroup, according to the defining proteins, as shown in a heatmap (Supplementary Fig. S5). Subsequently, each mouse was analyzed individually (Fig. 3 and Supplementary Fig. S8).

After the removal of clusters resulting from artifacts or uninterpretable clusters (see supplementary material), a total of 80,506 cells were analyzed in the 13 cores considered, with an average distribution of 6029 cells/core.

The normal mouse lung contained a majority of Alveolar Epithelial cells (67.9% ± 11.9), divided into AT1 (22% ± 5.8), AT2 (14% ± 9.7) and a population with co-expression of AT1 and AT2 markers, named transitional AT (32% ± 11.8). Each of the remaining constituents of the normal lung remained below the 10% value. Bronchial epithelial cells and goblet cells averaged 7% ± 7.7, endothelial cells were 3% ± 1.4, myofibroblasts/smooth muscle cells 2% ± 1.9, other stromal cells 0.7% ± 0.4. Resident hematopoietic cells were T lymphocytes (7% ± 1.5), B lymphocytes (3% ± 1.9), macrophages and dendritic cells (4% ± 1.9) and neutrophils (1% ± 1.2) (Fig. 3 and Supplementary Fig. S8).

**Figure 4:**
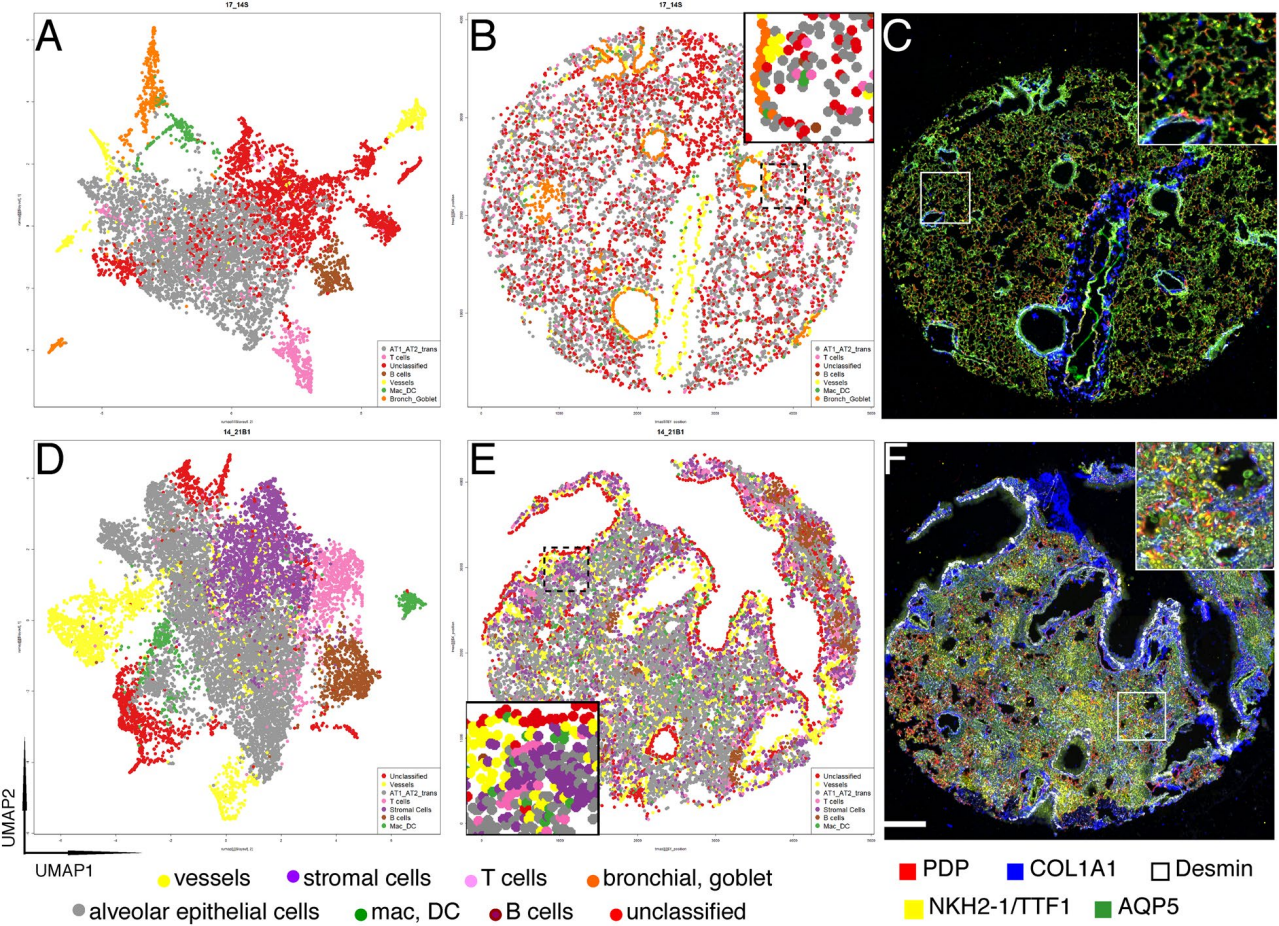
Spatial distribution of cell types. UMAP plots, spatial distribution of phenogroups and immunofluorescence images for representative untreated (**A**–**C**) and day 21 lungs (**D**–**F**). UMAP plots (**A**,**D**) are colored with the cell classification color (bottom legend). The same color-coded groups are plotted on the respective TMA core spatial coordinates (**B**,**E**). A detail is shown in the insets. Note in E a fibroblastic focus next to a bronchial lining population (red) and vessels. On the right five representative markers (color legend at the bottom) are shown in the whole core or in a high-magnification field (inset) (**C**,**F**). Scale bar 100 µm.

By plotting the cell types identified on the tissue coordinates, cell types composing the scaffold of the normal lung were organized in morphologically recognizable structures, while lung epithelial cells and other diffusely present cell types were evenly distributed across the tissue (Fig. 4 and supplementary Fig. S7), mimicking the image obtained by multicolor immunofluorescence (Fig. 4 and supplementary Fig. S4).

Single cells from mice treated with Bleomycin and examined at day 7, 14, 21 and 28 partially overlap with the control mice (Fig. 2), highlighting qualitative and quantitative changes in the lung population. By applying the same classification criteria used to classify the normal lung, one could observe a transient reduction in AT1 and a progressive decrease of AT2 pneumocytes (Fig. 3), largely due to changes in an AT2 subpopulation (transitional AT2, see supplementary Fig. S7 and S8). Stromal cells increased progressively, accompanied by a late increase of myofibroblasts/smooth muscle cells, mimicking the histopathologic accumulation of stroma (see supplementary Fig. S1). The remaining lung cells remained stable except for a late decrease of macrophages, neutrophils and B lymphocytes. Plasma cell markers were not included in the panel, thus we could not assess whether the decrease in B cells was due to absence or maturation.

The spatial distribution of the cell types revealed a crowded parenchyma and focally increased stromal foci, B cell and macrophage aggregations (Fig. 4).

A drug transmembrane transporter, P-gP, constitutively expressed in many cell types, was measured in lung cells identified by high dimensional analysis.

**Figure 5:**
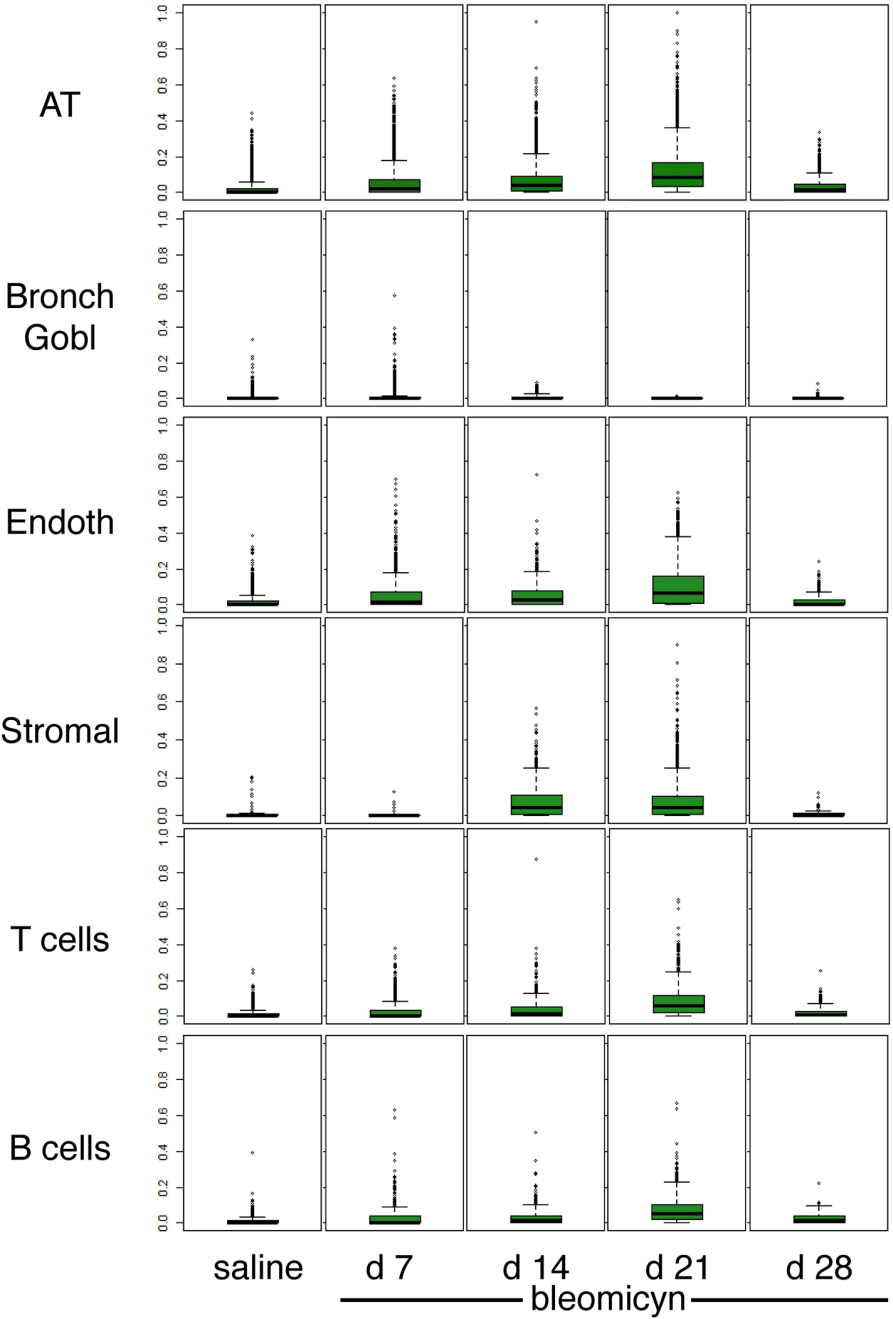
Multidrug transporter (P-gP) levels during Bleomycin treatment in selected cell types. The linear fluorescence levels (0–255), rescaled 0–1 of the multidrug transporter from all experimental animals are plotted as boxplots and outliers for each treatment group and for the selected cell types. AT = all alveolar epithelial cells; Bronch Gobl = bronchial cells and MUC5B + cells merged.

Increasing intensity signal was registered in all populations after Bleomycin treatment, except in bronchial cells (Fig. 5 and supplementary Fig. S9). The onset of the time-dependent increase was delayed in the stromal cell type (Fig. 5).

## Discussion

We have obtained an in situ spatial representation of the normal and of the fibrotic mouse lung by single cell classification via multiple antibody staining. Analogous to a single cell classification of murine (and human) lung cell component by single cell RNA sequencing^15–18^, here we show that the content of a normal or diseased organ such as the lung can be finely dissected at the single cell level, with two additional properties: spatial cell disposition is represented and proteins, instead of RNA, are assessed by a robust, cheap and versatile method.

With a rather limited number of validated antibodies, we can identify in situ the main cell types which are relevant for lung homeostasis and for the initiation and establishment of the Bleomycin-induced fibrosis: AT1, AT2, transitional AT1-AT2 pneumocytes, bronchial lining cells, vasculature, stromal cells, T and B lymphocytes, macrophages. In addition, we measured at the single cell level continuously expressed proteins such as a transcellular drug transporter, P-gP.

We measured with the same detail the changes occurring upon Bleomycin treatment over time, documenting a change in the AT1/AT2 ratio, the progressive accumulation of stromal cells and the asynchronous increase of P-gP expression.

Lastly, we included in the assay a high throughput method, the tissue microarray technology, after careful validation of the representativeness of tissue core size.

We suggest the use of a 2 mm TMA core as a minimum tissue area for the evaluation of tissue cell composition on mouse lung. We came to this conclusion by performing high-dimensional analysis as a validating tool, applicable to TMAs from tissues of various origin and composition and by taking into account the highest total cell number compatible with TMA sampling of the lung.

The most abundant population we found in normal tissue and also in BLM-treated mice is represented by epithelial cells.

Data which has been obtained by tissue dissociation and single cell sequencing of normal mouse lungs^19,20^ do not agree among themselves about the representation of the cell types, probably because of different pre-analytical dissociation methods and inherent selective loss of epithelial cells or enrichment of other cells. The drawbacks of the tissue dissociation methods are known^21^. By in-situ cell classification we can provide an unbiased estimate of the various lung cell components. In addition, by measuring the end product of the transcription and translation machinery, we complement the data provided by RNA sequencing.

Analogously to scRNA sequencing data, the alveolar epithelial cell populations do aggregate in closed contact in the bi-dimensional tSNE space, reflecting a continuum of phenotypes, interpretable as a transition from AT2 to AT1 cells. We tentatively identified a reproducible subset of AT2 cells bearing AT1 markers, which we dubbed “transitional AT” cells, analogously to a similar subset identified in Bleomycin-treated animals^15,17^. Whether the subset we identified by in-situ proteomics and the ones described in scRNAseq experiments are identical requires additional experiments with parallel analytical tools. We cannot completely exclude that two distinct AT1 and AT2 cells, closely spaced, are segmented as a cell with hybrid phenotype.

Collagen deposing stromal cells are the main actor in the fibrotic process produced in this experimental model. We could demonstrate an increase in stromal cells which parallels the histomorphologic changes. According to our data, these cells represent a minority of the lung population, to the point that are inconsistently demonstrated in the normal lung, at variance with data obtained by dissociation and RNA sequencing. Besides the differences in methods, as outlined before, stromal cells are underrepresented in our markers panel; in addition, a nuclear-based cell segmentation is not ideal to identify elongated cells, thus we may underestimate this cell subset, despite showing a treatment-dependent increase.

Histochemical stains do not discriminate the cell of origin for collagen deposition, nor if the collagen is deposited by pre-existing cells. On the other hand, we assess stromal cells individually and by intracellular markers, documenting a net increase in nucleated stromal cells upon treatment: it is thus not unexpected that the two represent non-identical assays, as demonstrated by the data.

The progressive decrease of the AT2 and particular of the transitional AT over time and the increase of stromal cells we have shown may highlight the key drivers of lung fibrosis in this mouse model: a progressive reduction of the alveolar lining repair by depleting local alveolar progenitors and surfactant producing cells, AT2, coupled with newly produced, collagen depositing fibroblasts. These results are novel and should be consolidated by independent experiments and increased sample numerosity.

The time-course expression of P-gP has been previously published for the same Bleomycin-induced mouse lung model^14^, with notable differences: the experiments were performed on male mice and the RNAs for the multidrug transporters were measured, both as whole tissue extracts and by in-situ hybridization. We have reproduced the data on male mice (see Supplementary data), and shown that female mice differ in the kinetic of the pathology but not in the type of histopathologic lung changes. In addition, by measuring the multidrug transporter protein at the single cell level on each and every cell type in the specimen, we have detailed a cell-type specific kinetics of P-gP expression, with some notable difference with the RNA data of Park et al.^14^.

Limitations of the study: We employed a limited number of antibodies, compared to the potentiality of the MILAN technique (over 100); this because there is a variety of reagents to choose from which is more limited than what is available for human FFPE tissue, particularly in terms of species origins of the antibodies. Being the MILAN technique based on multiple (3 or 4) unconjugated antibodies per round, we partially overcome these limitations by using mouse antibodies on mouse tissue, background free^5^. It has been shown previously however, that the discriminating power of the dimensionality reduction algorithms is so high, that even with a reduced number of diagnostic parameters, the cell types of interests can still be identified^22^ and this is the case for the present work.

An additional limitation of this study is the use of a rather unsophisticated image segmentation technique and manual cell type assignment for the phenogroups. Cell segmentation is one of the most challenging tasks for in situ transcriptomics and proteomics^23^ and efforts are ongoing to improve it for the mouse lung fibrosis model. In this model, elongated cells such as fibroblasts and stromal cells in general poorly fit cell identification via nuclear DAPI identification. This may be the reason why in normal lungs we occasionally found minimal or no fibroblast cell clusters, together with the small total amount, in common with cell suspension based studies^15–18^. Along the same lines, we found clusters containing markers of two distinct cells (e.g. epithelial and macrophages, epithelial and stroma) which we were forced to discard, because of the juxtaposition of elongated cells was not solvable with the cell segmentation strategy used, despite being clearly identifiable by the tissue spatial distribution (see supplementary data/discussion).

Lastly, despite the nucleus-centered segmentation strategy, which limits the sampling of nearby cells, signals of diffusible and/or extracellular proteins may leak into another “pseudo-cell”, providing a hybrid signature, but also important biologic information about closely adjacent cells.

In summary, we have shown proof of principle that mouse tissue, being a normal organ, a pathology model or a developing tissue, can be dissected on routinely processed material by in-situ high-dimensional proteomics and single cell bioinformatic analysis. This represents a powerful tool for pre-clinical studies such as drug discovery and novel treatments and can be integrated with other “omics” tools such as scRNA sequencing, in situ transcriptomics etc.^24^.

## Materials and methods

### Experimental animals and histology

A mouse model of Bleomycin-induced lung fibrosis, currently in use in our institution, has been previously published^25^ and has been approved by the internal AWB (Animal Welfare Body) of Chiesi Farmaceutici under protocol number: 841/2019-PR and comply with the European Directive 2010/63 UE, Italian D.Lgs 26/2014 and the revised “Guide for the Care and Use of Laboratory Animals” and the Animal Research: Reporting of In Vivo Experiments (ARRIVE) guidelines (www.arriveguidelines.org).

In brief it consists of the oropharyngeal BLM administration to 7–8 weeks old C57Bl/6 female mice of 15 µg/mouse at each day of treatment respectively. Animals were sacrificed at 7, 14, 21 and 28 days after the administration.

After the sacrifice, lungs were removed, formalin fixed and paraffin embedded (FFPE). Three serial sections, 5 μm thick were obtained for the stainings: Hematoxylin and Eosin, Masson’s trichrome (TM) and a multiplex immunostaining.

Whole-slide images were acquired by a NanoZoomer S-60 Digital slide scanner (NanoZoomer S60, Hamamatsu, Japan) at 20× magnification. Fibrotic lung injury was assessed histologically through the Ashcroft scoring system^26,27^ on whole parenchyma by two independent researchers (blinded to the experimental design). Detailed description and histomorphometric characterization are included in the Supplementary methods and in Supplementary Figure S1. The most relevant areas on the slide were marked for subsequent tissue microarray construction (see below).

### Tissue microarray design and construction

For the TMA preparation, we used FFPE tissue blocks of lungs from BLM-treated female mice. 13 cases of treated and control lungs were selected; cores were extracted, selecting specific regions representing normal lung tissue with alveolar parenchyma, bronchioles and vessel for saline samples and fibroproliferative foci with its alterations for Bleomycin ones from the C57BL/6 female mice FFPE lungs of BLM and saline treated mice across four time points (7, 14, 21 and 28 days, see Supplementary Table S1). The TMA was constructed with a Tissue Microarrayer Galileo CK4500 (Tissue Microarrayer Model TMA Galileo CK4500; Integrated Systems Engineering srl, Milano, Italy) using Galileo Software to match the annotated tissue on histological slide with their corresponding areas on the surface of the paraffin donor blocks. After the core transfer in the recipient block, to allow the samples to be properly embedded into the block, the TMA was incubated for 24 h at + 38 °C. Finally, 5 μm thick serial sections were cut from the TMA with a rotary microtome (Slee Cut 6062, Slee Medical, Mainz, Germany) and placed on Polysine adhesion glass slides (Thermo Fisher Scientific). Routine histology was performed as described previously^25^.

In order to select the appropriate Tissue Microarray (TMA) core dimension, after applying a high-plex MILAN staining method^6^ on the whole slide of a mouse lung, we selected 4 virtual cores (with ImageJ) of 1 mm and 4 of 2 mm in diameter. Thus we run single cell analysis as previously described on 8 cores and on a whole slide and we compare the results. The level of adequacy of tissue portion was set when all main cell populations found in whole tissue by clustering analysis (Rphenograph package) were rediscovered in cores. Moreover, the 2 mm core better includes the patchy distribution of fibroproliferative foci within the parenchyma.

### Highplex immunofluorescence

Lung samples were processed as per the Milan (Multiple Iterative Labeling by Antibody Neodeposition) protocol^6^ modified for mouse FFPE tissue staining. The protocol consists in the cyclic application of primary antibodies (Table 1) raised in multiple species, including mouse^5^, applied on the same section, nuclear staining with DAPI, autofluorescence subtraction^28^. For each round, the stained slides were scanned and saved as digital images using a multichannel fluorescence acquisition instrument (NanoZoomer S60, Hamamatsu, Japan). The antibody stripping preceded a subsequent staining round. A multichannel fluorescence acquisition was performed after each stripping, in order to check the complete antibody removal.

In order to generate an antibody panel which would produce single cell lung classification, we mined existing single cell RNA sequencing (scRNAseq) studies^19,20^ for highly expressed, lineage-restricted messages which would correspond to proteins against which antibodies would be available. The staining sequence design and antibodies validation and specifications were provided in Table 1. The methods used for the antibody selection and validation are reported in Supplementary methods.

Sections were incubated overnight with primary antibodies, which were then revealed by secondary fluorochrome-tagged antibodies; both isotype control and omission of primary have been performed as negative control. Sequential stripping was obtained with a beta-mercaptoethanol and sodium dodecyl sulphate mix^28^.

Mouse tissue preservation after each of multiple staining and stripping rounds showed no or negligible cell loss (Supplementary Figs. S2 and S3), as measured by DAPI nuclear stain and quantification on four other tissues (kidney, liver, lung and heart)*.*

### Single cell analysis

After the stainings were acquired, digital slide images (.ndpi) were imported as .tiff and registered with the AMICO software^29^, based on Fiji. All DAPI images from different staining rounds were registered together and their coordinates of rotation and translation were used to align the individual marker images of each round. Once all images were aligned, autofluorescence was subtracted from FITC and TRITC channels.

DAPI stainings were used for cell segmentation with Fiji (Threshold Default -Watershed_Analize particles and Count mask creation), where each nucleus and surrounding cytoplasm is identified by a single Region of Interest (ROI).

Cell segmentation based on DAPI or other markers is a bioinformatic approximation to physical single cell isolation, while maintaining in situ cell position and microenvironment location. The term “single cell” will be used throughout for this approximation.

Mean intensity value of all markers within each segmented cell and spatial coordinates of centroids of nuclei were recorded together in a .csv file.

Then .csv files were uploaded to the R Studio software (version 1.4) for a more detailed analysis. Rtsne^30^, umap^31^ and Rphenograph^32^ (n = 30) algorithms (R packages) were used respectively for dimensionality reduction and clustering of data. UMAP plots were decorated with single individual relevant marker intensity to evaluate the distribution among phenogroups.

A single tSNE plot from homogeneous groups of animals was used to compare treatments versus control (Fig. 2), exclude an experimental batch effect (Fig. 2) and explore the cell type of the clusters identified by Phenograph with a comprehensive heatmap (Supplementary Fig. S5).

Subsequently, the samples were analyzed individually.

Clusters obtained were further explored via: (i) individual cell immunoprofiling through dedicated hierarchical clustering heatmaps as published^33^ (Supplementary Fig. S6), (ii) visualization of the spatial distribution on tissue (Figs. 4 and S7). Clusters which satisfy both criteria were manually classified according to the expressions of key markers (Table 1).

Major cell types were created by merging clusters with a similar phenotype, after removing artifacts. Main types were organized as follows: (1) Alveolar Epithelial cells subdivided in AT1, AT2 or transitional AT1-AT2 subpopulations, (2) Bronchial and Goblet Epithelial cells, (3) Macrophage cells, (4) B cell, (5) T cells, (6) Vasculature and (7) Stromal cells, Neutrophils (8). For graphic spatial representation, all epithelial cells were grouped in a single entity (Alveolar Epithelial Cells).

Phenoclusters for which no coherent phenotype was identifiable were excluded from the analysis (see supplementary material for further information).

The transporter expression was analyzed by measuring the mean of the intensity of the signal (on 8-bit grayscale images, values 0–255) within each main type.

### Ethics approval and consent to participate

All the animal experiments were performed according to the ARRIVE guidelines (www.arriveguidelines.org) and European Directive 2010/63 UE, Italian D.Lgs 26/2014, and associated guidelines.

## Supplementary Information

Supplementary Information.
