# Supplementary material for: The normal and fibrotic mouse lung classified by spatial proteomic analysis
Source: Sci Rep. 2022 May 24;12:8742. doi: 10.1038/s41598-022-12738-9 (PMC9130283; doi:10.1038/s41598-022-12738-9)
Supplement: Supplementary file 1 — Supplementary Information. [file 41598_2022_12738_MOESM1_ESM.docx]

**Supplementary Materials**

**THE NORMAL AND FIBROTIC MOUSE LUNG CLASSIFIED BY SPATIAL PROTEOMIC ANALYSIS**

Roberta Ciccimarra^1^*, Maddalena M. Bolognesi^2^*, Matteo Zoboli^1^, Giorgio Cattoretti^2^, Franco F. Stellari^3#^, Francesca Ravanetti^1^

^1^Department of Veterinary Science, Università di Parma, Parma, Italy

^2^Department of Medicine and Surgery, Università di Milano-Bicocca, Monza, Italy

^3^Corporate Preclinical R&D, Chiesi Farmaceutici S.p.A., Parma, Italy

^*^R. Ciccimarra and M. Bolognesi contributed equally to this work.

**Materials and Methods**

**Experimental animals**

Female and Male inbred C57Bl/6 (mice were purchased from Envigo, Italy (San Pietro al Natisone, Udine, Italy). Prior to use, the animals were acclimatized for at least 5 days to the local vivarium conditions (room temperature: 20–24°C; relative humidity: 40–70%; 12-h light–dark cycle), having free access to standard rodent chow and softened tap water. From day 0, every other day, mice were given high calories dietary supplement (Recovery gel, Dietgel, ClearH2O, Westbrook, ME, USA) and sterile sunflower seeds in addition to the standard rodent chow.

BLM dosage was 25 µg/mouse for female, 15 µg/mouse for males at each day of treatment respectively. For the BLM administration mice were lightly anesthetized with 2.5% isoflurane delivered in a box and bleomycin hydrochloride (BAXTER) was administered via oropharyngeal (OA) using a micropipette positioned on the intubation platform, hanging them by their incisors placed on the wire, the tongue was pulled out with forceps, and, using a small laryngoscope, the liquid was placed onto the distal part of the oropharynx with a micropipette while the nose was gently closed. This procedure was performed at day 0 and 4.

After the euthanasia, lungs were removed, inflated with a cannula through the trachea by gentle infusion with 0.6 ml of 10% neutral-buffered formalin and fixed for 24 h. Lungs were dehydrated in graded ethanol series, clarified in xylene and paraffin embedded.

**Histology and histomorphometry**

Fibrotic lung injury was assessed histologically through the Ashcroft scoring system ^1,2^ by two independent researchers blinded to the experimental design. For Ashcroft score analysis the whole lung parenchyma was considered for the analysis at the magnification of 20X on the virtual slide. To improve its informativeness, the Ashcroft score was subdivided into three main categories: mild (0-3), moderate (4) and severe (5-8), their frequency distribution was then analyzed. The collagen content was measured on TM-stained lung sections using the image analysis software NIS-Elements AR 3.1 (Nikon, Tokyo, Japan) applying a green threshold detected on the Light Green stained collagen fibers. As an indirect parameter of fibrosis, the alveolar air area was quantified through a white based threshold, using the same software and ROIs applied for the collagen content. The Collagen content and Air Area were expressed as a percentage of area (μm^2^) occupied by Collagen/Air referred to the lung total area (μm^2^) within the ROI. Bronchi and blood vessels have been excluded from the analysis and from the ROI area. (Supplementary Fig. S1).

**Antibody selection and validation**

The selection of antibodies was made within those validated, from the supplier or in house, according to the criteria published by previous papers ^3,4^ if both the homology at the peptide level was above 95% and the tissue distribution matched that expected and already predicted by RNA Seq data.

The examination of online antibody listings from commercial suppliers starts from web-based resources Biocompare ([www.biocompare.com/Antibodies/](http://www.biocompare.com/Antibodies/)) and Antibody Resource Page ([www.antibodyresource.com](http://www.antibodyresource.com)). Other specific public data bank that contained the comprehensive and freely accessible resource of proteins sequence and functional information were consulted. Expression analysis datasets were obtained from BioGPS database (http://Biogps.org/#goto) and Uniprot database (<https://www.uniprot.org/>), protein interaction networks were obtained from Gene Card human gene database (<http://www.genecards.org/>).

**Highplex immunofluorescence**

In order to evaluate tissue conservation among rounds, a cell number count, based on DAPI segmentation, was obtained for each core. In Supplementary figure S2 we show the comparison among numbers of cells segmented in each round. Variations were due to occasional cells loss for mechanical unexpected events (scratching). No statistically significant differences were revealed among rounds. Separately we also evaluated tissue-specific cell loss on mouse kidney, liver, lung and heart (4 rounds of stripping) (Supplementary Fig. S3)

Cell count based on DAPI segmentation demonstrated tissue-preservation among rounds and organs, confirming the reproducibility of MILAN technique on mouse FFPE tissue. In supplementary Fig. S4 we show a representative tissue area stained with 22 antibodies in sequential reactions, perfectly conserved among rounds.

**Cell classification**

Phenoclusters were assigned to a given cell type based on: i) positive lineage-defining markers (see Table 1 and Supplementary Fig. S5 and S6) in a coordinated fashion; ii) spatial distribution coherent with the putative cell type (see Fig. 4 and Supplementary Fig. S6 and S7). Due to a close juxtaposition of cells of different origin (e.g. alveolar epithelial and fibroblasts, alveolar macrophages and epithelial cells) and the limitation of a cell segmentation based on an expanded nuclear mask, the spillover of signals from two adjacent unrelated cells was observed but did not disqualify the lineage assignment. Failure to assign a cell type to a phenocluster was due to artifacts (focal tissue loss down the staining sequence, unresolved autofluorescent spots: “junk” clusters) or phenoclusters validated by only one of the two criteria mentioned above (unclassifiable clusters). No distinction was made between these two scenarios in the analysis and these were excluded from the calculation.

Alveolar epithelial cells were resolved in three groups in most if not all cases (Supplementary Fig. S8); the two closely positioned groups in the UMAP plots (Supplementary Fig. S7) shared an AT2 phenotype (e.g. NKX2-1/TTF1), but one had additional AT1 markers and this latter was therefore named “transitional AT”.

**Multidrug transporter (PgP) expression evaluation**.

The mean intensity of PgP expression was quantified per cell type per sample and plotted (Fig. 5). In addition, a visual representation in immunofluorescence was rendered on representative samples at different time points (Supplementary Fig. S9).

**Supplementary Table legend**

**Supplementary Table 1**

Cell count for the control and experimental mice, assigned to each cell type.

**Supplementary Figures legends**

**
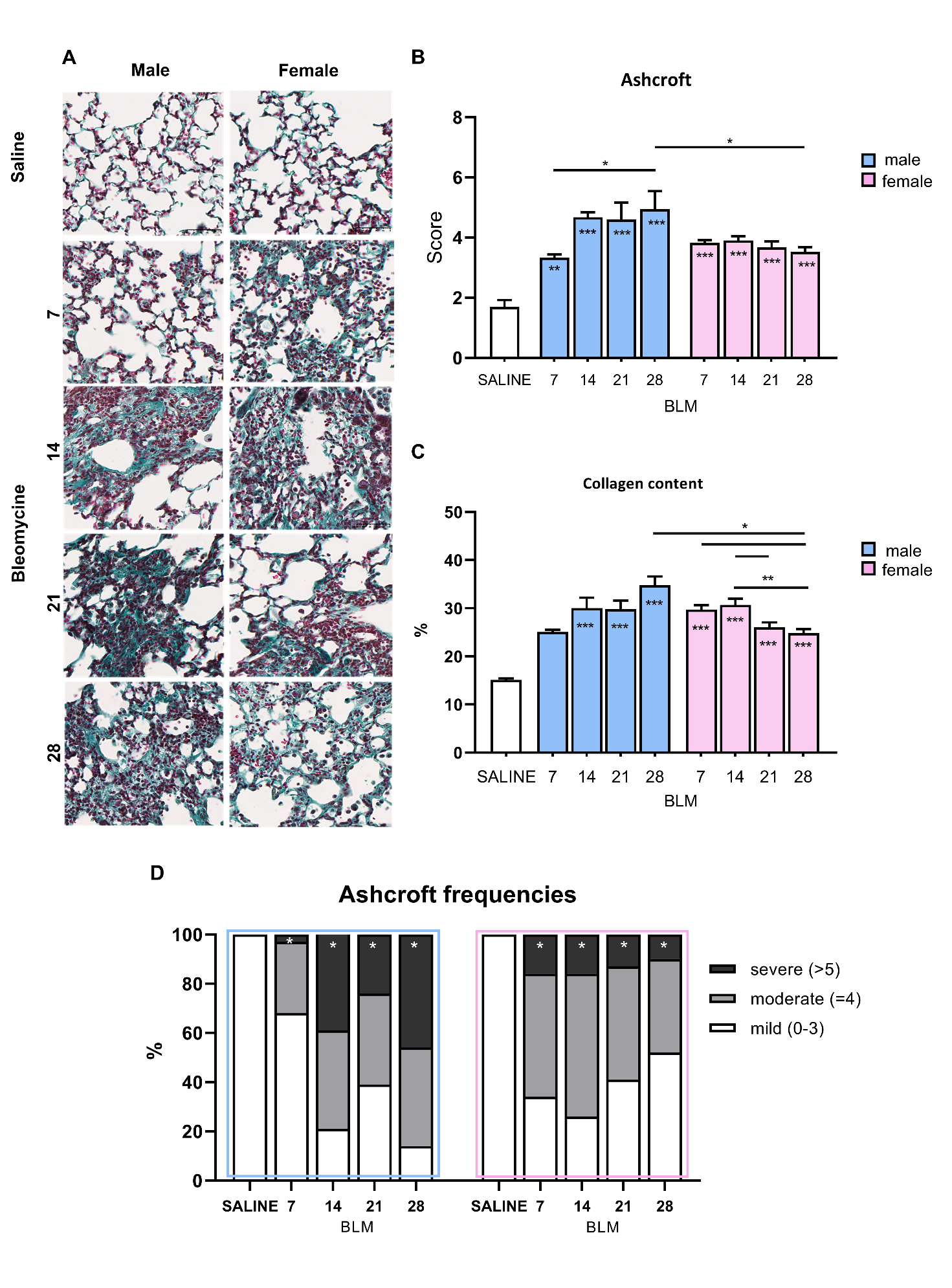
**

**Supplementary Figure S1**

Histology and histomorphometry results.

(A) Representative histological microphotographs of male and female mice lung parenchyma stained with Masson Trichrome at 40X magnification (scale bar 50um). The panel shows, for both genders, the BLM induced fibroproliferative alteration of lung parenchyma during the time course. Collagen based matrix is stained in green, the cellular component in violet. (B) Ashcroft score plotted as histograms. (C) Collagen content quantification expressed as a percentage. Asterisks indicate significance of BLM groups compared to the vehicle. (D) Frequency distribution of the Ashcroft score values grouped as mild (0 –3), moderate (4), and severe (5). Asterisks within the bars indicate significant differences from saline; asterisks above the horizontal lines indicate significant differences of the severe grade between genders at the same time point (***P < 0.001; **P < 0.002; *P < 0.33).


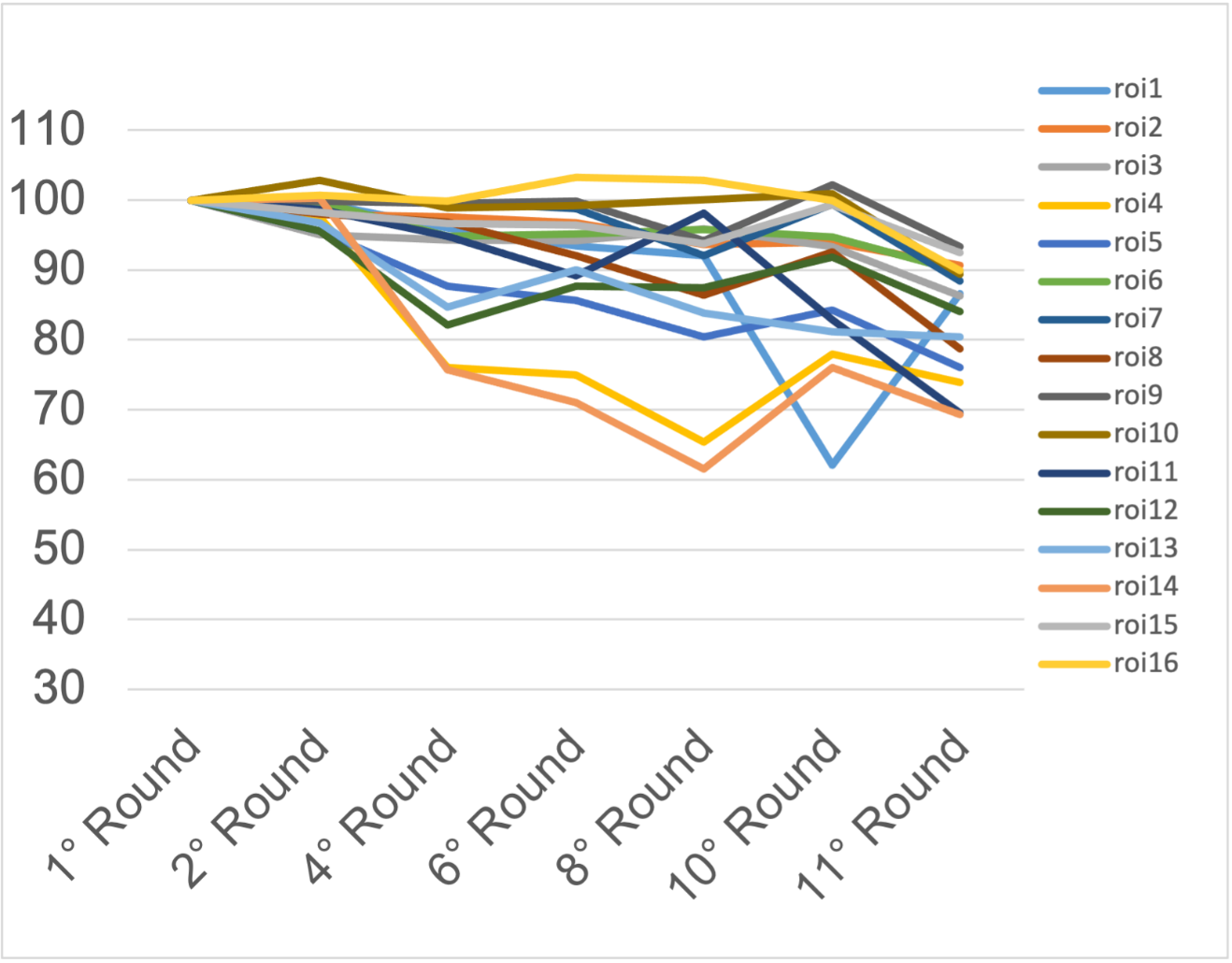
**Supplementary Figure S2**


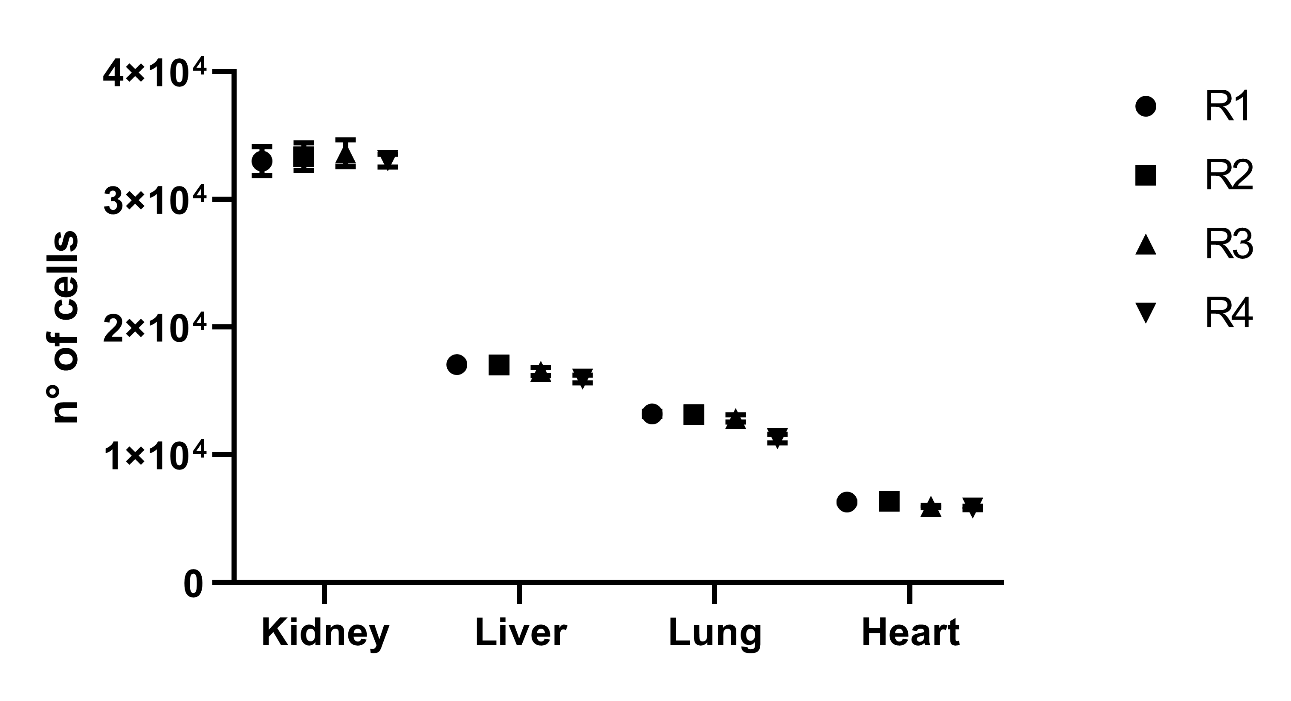
Tissue conservation among 11 rounds in lung TMA cores. Initial counts for each core = 100%.

**Supplementary Figure S3**

Tissue conservation among four rounds in multiorgans TMA. Tissue-type conservation among rounds: each dot represents the average cell number in three cores of 2 mm in diameter. No statistical differences were observed. (Scale bar 100 um).

**
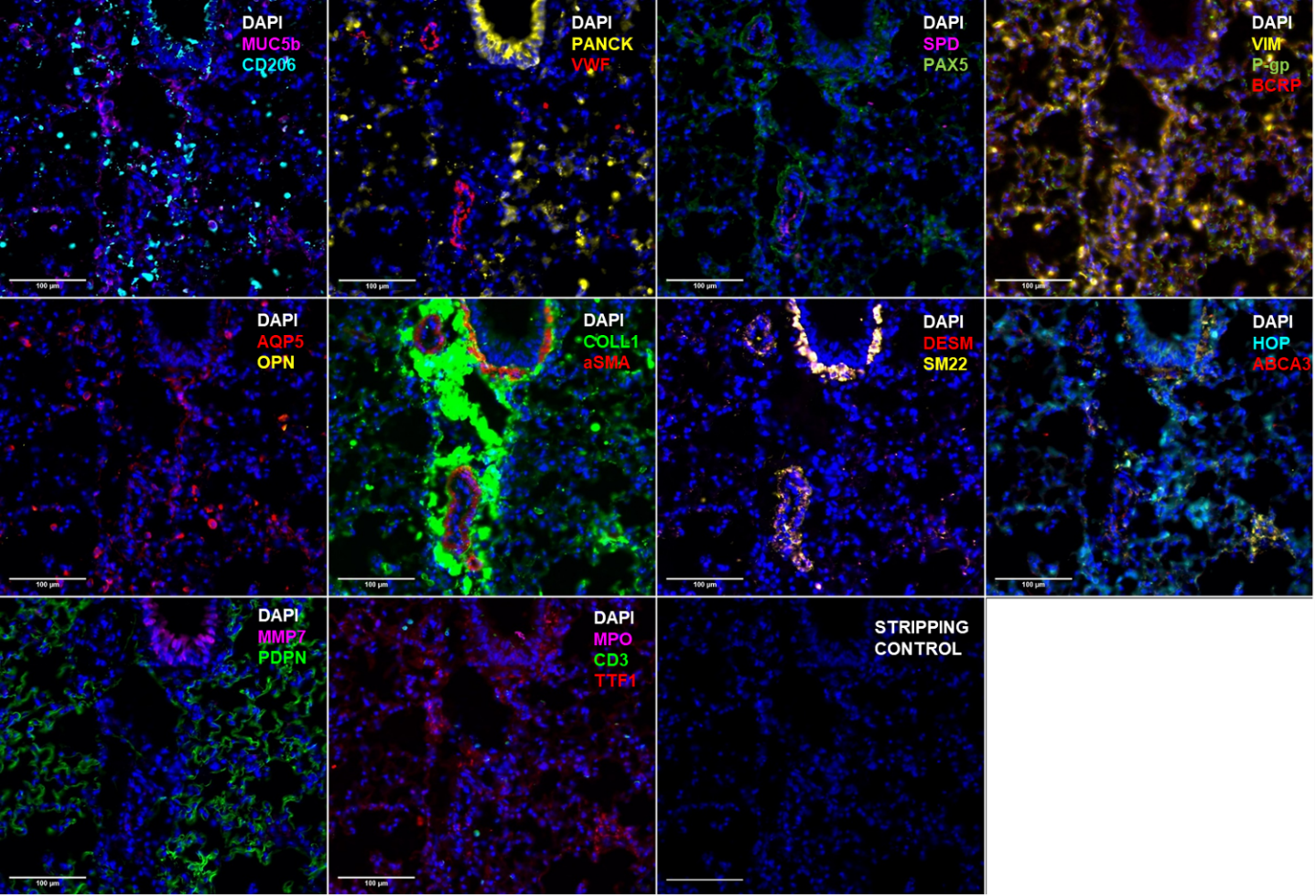
**

**Supplementary Figure S4**

Highplex Immunofluorescence with 22 antibodies in 11 sequential rounds of IF staining.

Microphotographs of multiple markers related to different cell histotypes. Pseudo colors were used to show distinct signal and localization. The last microphotograph is an example of stripping control that was applied for each round and filter. (Scale bar 100 um).


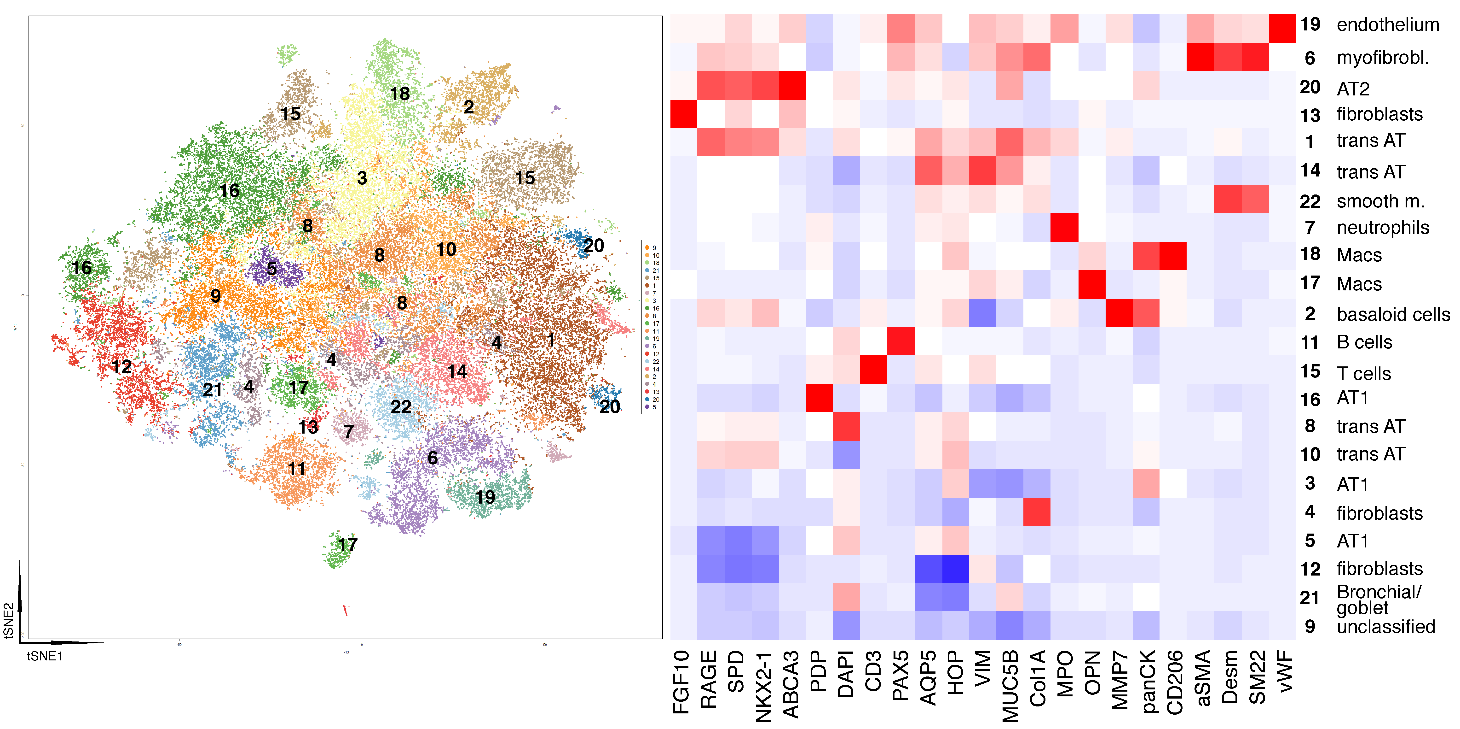


**Supplementary Figure S5**

Distribution of selected markers and cell types in dimensionality-reduced 2-dimensional space (tSNE) plots.

The mean marker expression of all markers is shown as an heatmap (right) for each phenogroup identified in the tSNE plot (left). A lineage assignment is shown on the right of the heatmap, next to a phenogroup number, which is placed on the tSNE plot. Color code for the heatmap: red = positive, blue = negative.


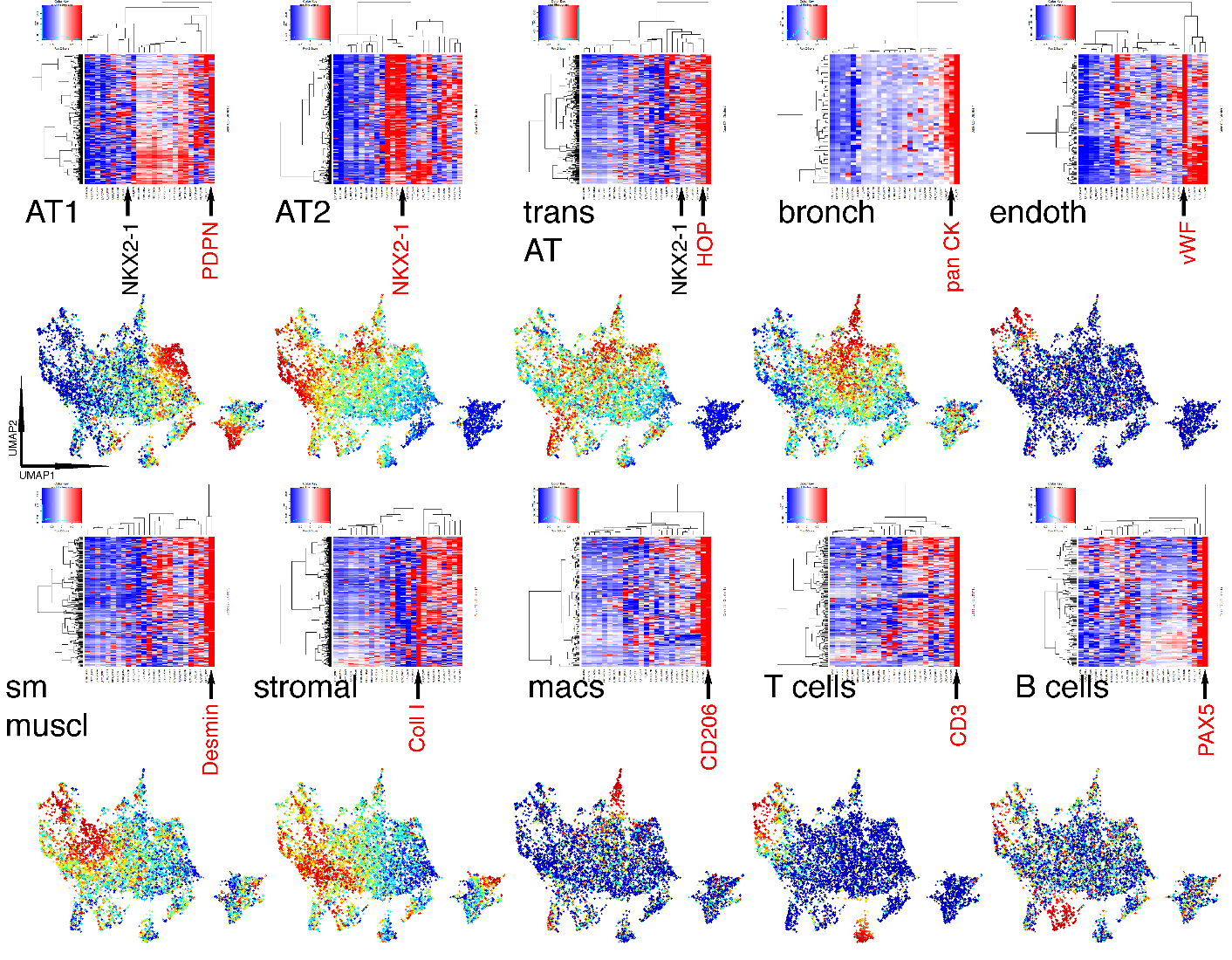


**Supplementary Figure S6**

Cell type classification of phenoclusters.

For each cell type, the heatmap of a representative phenocluster, one defining cell marker (in red) and the marker distribution on the UMAP of the core is shown. For alveolar cell types (AT1, AT2 and trans AT) the column of an AT2 marker, NKX2-1, is arrowed, to highlight the phenotype of the three epithelial cell types. A day 21 bleomycin mouse is shown.

**
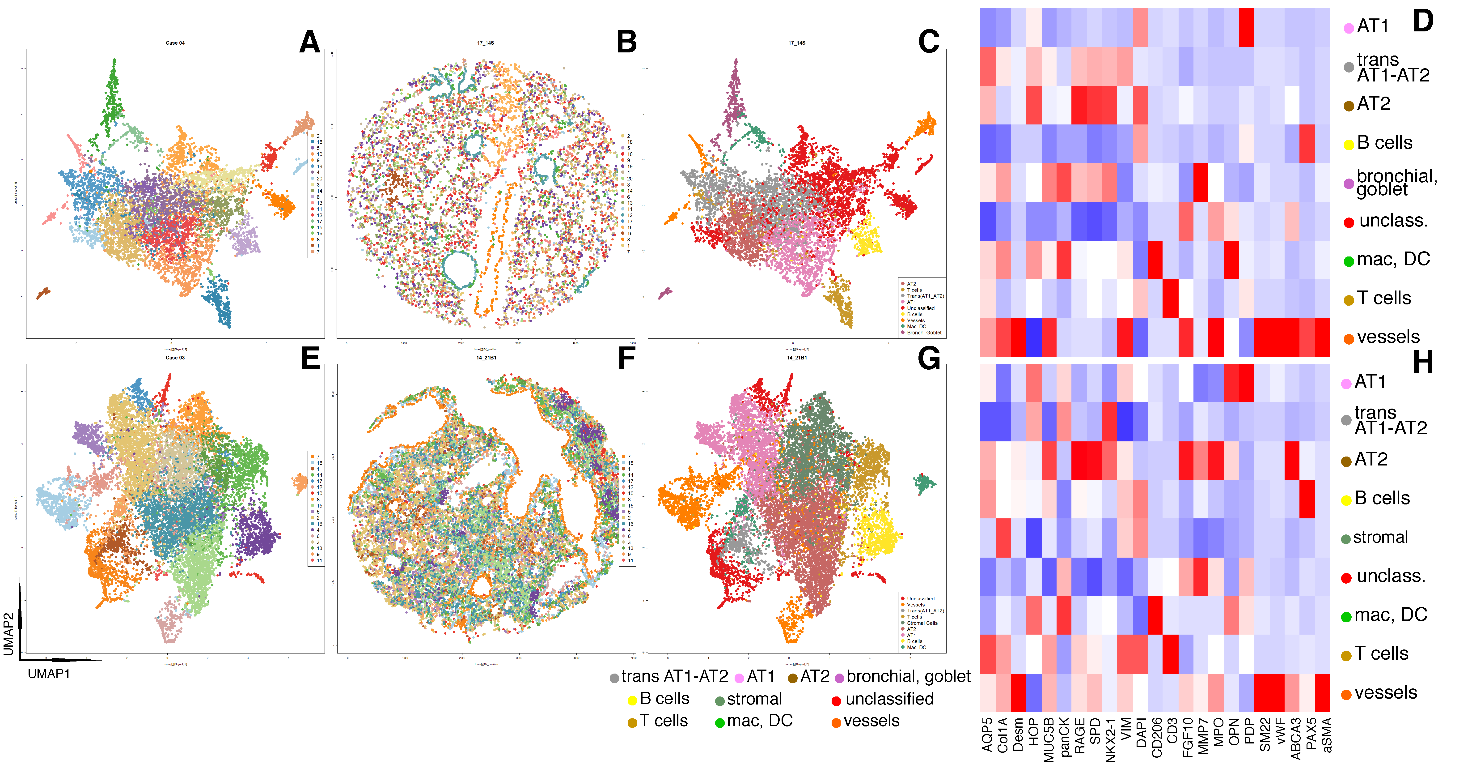
**

**Supplementary Figure S7**

Phenocluster composition and spatial distribution.

The phenoclusters of a control (A-D) and of a day 21 bleomycin mouse (E-H) are shown superimposed on a UMAP plot (A, E) and on the tissue core (B, F). The phenoclusters are re-classified (C, G) according to the cell type as in Figure 4, however AT1, AT2 and transitional AT are plotted separately, in order to show the reciprocal relationships in the UMAP plot. The heatmaps of the mean expression of each marker (bottom row in H) in both cases (D, H) is shown at the right.

**
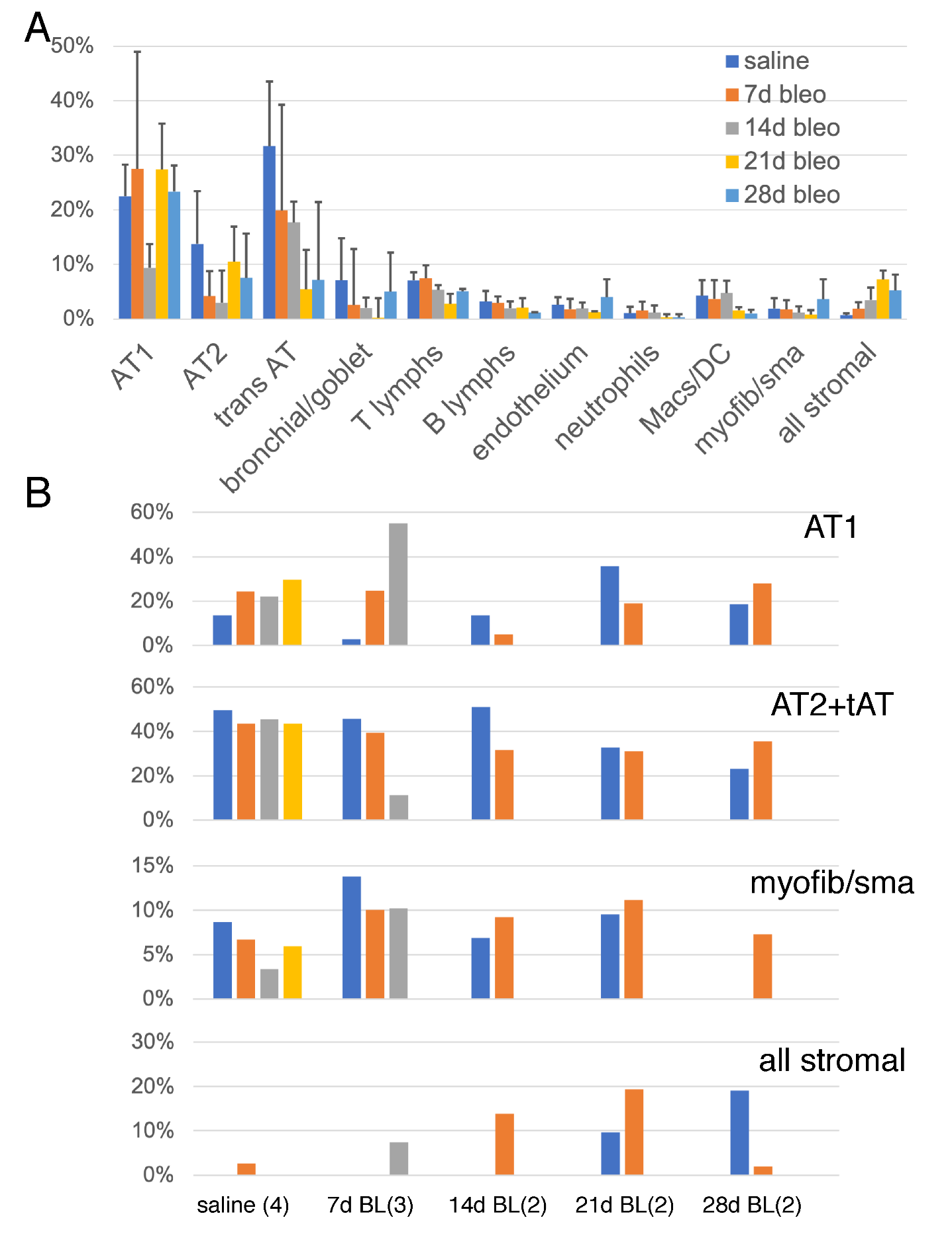
**

**Supplementary Figure S8**

Classification and frequency of cell types in controls and treated mice.

A: The cell types identified in the lungs and their frequency ±SD are shown. Trans AT = transitional AT1/AT2; bronchial/goblet = bronchial cells and MUC5B cells merged; all stromal = phenoclusters with mesenchymal markers merged. For details see supplementary Table 1

B: individual mice variations across the treatments are shown for four cell types. The number of mice is indicated in parenthesis after each treatment point.


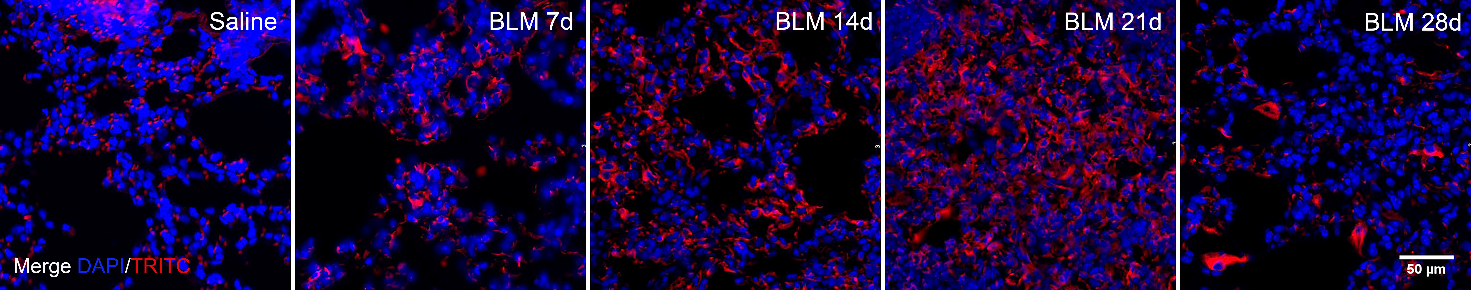


**Supplementary Figure S9**

Immunofluorescence staining of P-gp.

Immunofluorescence microphotographs of lung parenchyma and marked P-gp cells, shown through different time points (20X Magnification, Scale bar show 50 um). The nuclei were visualized using DAPI (blue) and P-gp was stained in TRITC (red).

**Supplementary References**

1 Ashcroft, T., Simpson, J. M. & Timbrell, V. Simple method of estimating severity of pulmonary fibrosis on a numerical scale. *J Clin Pathol* **41**, 467-470, doi:10.1136/jcp.41.4.467 (1988).

2 Hubner, R. H. *et al.* Standardized quantification of pulmonary fibrosis in histological samples. *Biotechniques* **44**, 507-511, 514-507, doi:10.2144/000112729 (2008).

3 Bolognesi, M. M. *et al.* Antibodies validated for routinely processed tissues stain frozen sections unpredictably. *Biotechniques* **70**, 137-148, doi:10.2144/btn-2020-0149 (2021).

4 Scalia, C. R. *et al.* Epitope recognition in the human-pig comparison model on fixed and embedded material. *J Histochem Cytochem* **63**, 805-822, doi:10.1369/0022155415597738 (2015).
